# Unmet Needs in Children With Attention Deficit Hyperactivity Disorder—Can Transcranial Direct Current Stimulation Fill the Gap? Promises and Ethical Challenges

**DOI:** 10.3389/fpsyt.2019.00334

**Published:** 2019-05-16

**Authors:** Anna Sierawska, Alexander Prehn-Kristensen, Vera Moliadze, Kerstin Krauel, Rafal Nowak, Christine M. Freitag, Michael Siniatchkin, Alena Buyx

**Affiliations:** ^1^Division of Biomedical Ethics, Institute of Experimental Medicine, Christian-Albrechts-University of Kiel, Kiel, Germany; ^2^Department of Child and Adolescent Psychiatry and Psychotherapy, Centre for Integrative Psychiatry, School of Medicine, Christian-Albrechts-University of Kiel, Kiel, Germany; ^3^Institute of Medical Psychology and Medical Sociology, Christian-Albrechts-University of Kiel, Kiel, Germany; ^4^Department of Child and Adolescent Psychiatry and Psychotherapy, University of Magdeburg, Magdeburg, Germany; ^5^Neuroelectrics Barcelona, Barcelona, Spain; ^6^Department of Child and Adolescent Psychiatry, Psychosomatics and Psychotherapy, University Hospital Frankfurt, Goethe University, Frankfurt, Germany; ^7^Clinic for Child and Adolescent Psychiatry and Psychotherapy, Medical Center Bethel, Bielefeld, Germany; ^8^Institute for History and Ethics in Medicine Medical School, Technical University of Munich, Munich, Germany

**Keywords:** tDCS—transcranial direct current stimulation, ethics, pediatric research, ADHD (attention deficit and hyperactivity disorder), noninvasive brain stimulation (NIBS)

## Abstract

Attention deficit hyperactivity disorder (ADHD) is a disorder most frequently diagnosed in children and adolescents. Although ADHD can be effectively treated with psychostimulants, a significant proportion of patients discontinue treatment because of adverse events or insufficient improvement of symptoms. In addition, cognitive abilities that are frequently impaired in ADHD are not directly targeted by medication. Therefore, additional treatment options, especially to improve cognitive abilities, are needed. Because of its relatively easy application, well-established safety, and low cost, transcranial direct current stimulation (tDCS) is a promising additional treatment option. Further research is needed to establish efficacy and to integrate this treatment into the clinical routine. In particular, limited evidence regarding the use of tDCS in children, lack of clear translational guidelines, and general challenges in conducting research with vulnerable populations pose a number of practical and ethical challenges to tDCS intervention studies. In this paper, we identify and discuss ethical issues related to research on tDCS and its potential therapeutic use for ADHD in children and adolescents. Relevant ethical issues in the tDCS research for pediatric ADHD center on safety, risk/benefit ratio, information and consent, labeling problems, and nonmedical use. Following an analysis of these issues, we developed a list of recommendations that can guide clinicians and researchers in conducting ethically sound research on tDCS in pediatric ADHD.

## Introduction

Attention deficit hyperactivity disorder (ADHD) is a neuropsychiatric disorder characterized by developmentally inappropriate levels of hyperactivity, impulsivity, and inattention (1). With a prevalence of about 5%, ADHD is one of the most prevalent childhood disorders, often associated with oppositional-defiant conduct, tics, anxiety, and mood disorders (2). Furthermore, a range of adverse long-term outcomes, including increased rates of accidents and injury, poor educational achievements, increased risk of substance abuse, and criminal activity, emphasize the individual and societal burden of ADHD (2, 3). Treatment mainly involves a combination of stimulant medication (4) and behavioral therapy (5). While the treatment with stimulants is characterized by high effect sizes (>1), it comes with a number of adverse events (6). These include a decreased appetite, sleep problems, headaches, nausea, and delayed growth. Patients are known to discontinue treatment because of these adverse events (7). In addition, in some children and adolescents, the medication shows an insufficient improvement of symptoms, especially of cognitive and motor inhibition or inattention symptoms (8). Alternative treatment options such as behavioral therapy, neurofeedback, cognitive training, as well as dietary interventions have been studied (9). However, these are characterized by small effect sizes and are time consuming, requiring high motivation and compliance of patients and their families (5).

There is thus a need to develop additional treatment options for children and adolescents with ADHD. Transcranial direct current stimulation (tDCS) is a new treatment option that aims at improving cognitive as well as behavioral symptoms. TDCS is a brain stimulation technique. It applies a weak constant direct current between head-mounted electrodes, which affects relatively large cortical areas. The mechanisms are electrode-dependent and involve either membrane depolarization or hyperpolarization (10, 11) [for a review, see Ref. (12)]. TDCS is capable of inducing changes in neuronal membrane potentials in a polarity-dependent manner. It has been shown that the effects of tDCS are induced by membrane depolarization (anodal) and hyperpolarization (cathodal) (10, 13). Therefore, tDCS may prime the system by increasing or decreasing the excitability of the system, or by increasing or decreasing the threshold response [see Ref. (14)]. TDCS is regarded as a noninvasive brain stimulation in comparison to, e.g., deep brain stimulation (DBS), which involves neurosurgical procedures (15).

However, as with many novel medical interventions, it also comes with a number of ethical challenges.

Building on a short description of current evidence of tDCS as a treatment option for pediatric ADHD, we discuss the ethical implications of researching this technology for clinical translation. Based on the results of this analysis, we present preliminary guidelines to enable high ethical standards for future tDCS studies in children and adolescents.

## Methods

A systematic literature search was conducted, based on the methodology from systematic reviews of reasons (14). This included a broad database selection (PubMed/MEDLINE, PsycInfo, PhilIndex) and database-specific search strings with relevant keywords. Keywords were derived from the areas of “ethics,” “social,” “pediatric research,” “ADHD,” “neuropsychiatric disorders,” “brain stimulation,” and “tDCS.” Papers of all types were included in the analysis, ranging from articles providing a purely theoretical reflection to reports of empirical studies of ethical aspects, to policy proposals. Papers were excluded if they did not discuss any ethical or social issues specifically, such as when these where only mentioned in passing.

Ethical and social issues were then grouped according to the normative principles from established frameworks of research ethics (16, 17) and approaches to ethical innovation (18). Issues were compared to mainstream interpretations of the ethical and social principles from these frameworks and discussed in detail. The ethical analysis was thus inspired by the background ethical theory of reflective equilibrium, which seeks to establish coherence between a variety of established ethical principles (19, 20).

### Transcranial Direct Current Stimulation—Clinical and Societal Aspects

Several studies on tDCS have been performed in adults, but only ∼5% of all published papers described effects of tDCS in children and adolescents (21). Recent results suggest that tDCS is a promising additional treatment for pediatric ADHD (23, 24) [for review, see Ref. (25)], with some evidence that anodal tDCS can reduce clinical symptoms in children and adults suffering from ADHD. In particular, symptoms of inattention were reduced by tDCS (22, 23).

Additionally, in pediatric ADHD, tDCS did not only influence connectivity of neuronal networks that are involved in cognitive performance (24, 25) but also improved cognitive performance itself, such as inhibitory control (26–28) and attentional processes (26).

TDCS has been reported to show only limited adverse events in children, adolescents, and adults (21, 29, 30). The fact that the technology is inexpensive further renders tDCS an attractive tool to explore in research and ultimately, to use in treatment in children with neuropsychological disorders, including ADHD. It also has the potential of being used as a portable device at home by patients (31, 32).

In addition to research interest, tDCS has been gaining attention within the general population (33). The interest in nonmedical application of tDCS could be explained by media hype around this technique (34), by emerging online forums that discuss the use of “do it yourself” (DIY) devices, and by easy access to information about the application of tDCS (35). Some concerns about the unregulated, nonclinical use of tDCS, especially regarding cognitive enhancement, have been raised within the scientific community (36). However, there is still little guidance for tDCS researchers and, eventually, healthcare professionals on how to approach this issue.

Experts in the field have acknowledged the need of further regulations when applying tDCS in order to minimize ethical challenges for researchers and clinicians working with tDCS and to enable effective clinical trials (37). Some guidelines were formulated to support facilitating better clinical applications, including calls for ensuring safety by the monitoring and reporting of adverse effects and by acknowledging the differences between age groups (29). Similar issues were presented in another document, which stressed the importance of education of healthcare professionals and patients about tDCS (38). However, available recommendations mainly focus on the technical and practical issues when conducting clinical trials, or on information for manufacturers (39); their focus is also on studies with tDCS in general, and in adult populations (29). No clear guidance exists on how to conduct trials with tDCS in pediatric populations with neuropsychiatric disorders such as ADHD, and how to make sure that these trials help translate results into practice in an ethical manner.

### Ethical Challenges in the Development of Transcranial Direct Current Stimulation Treatment for Children With Attention Deficit Hyperactivity Disorder

The need to protect vulnerable groups in general and children in particular in research can sometimes lead to a vicious circle: for many treatments, evidence does not exist to initially establish, e.g., relevant safety thresholds. However, in order for research on a vulnerable group to be conducted, any relevant risks must be specified and ideally minimized, to satisfy the usual standards of minimal risk and minimal burden when conducting research in children and adolescents (16, 17). Because long-term evidence in tDCS is not available, this can be difficult to do, leading to difficulties in getting study approval, and thus, fewer studies, which then leads to a lack of evidence, and so on. This situation is compounded in children with ADHD, who are not only minors but also vulnerable due to the disorder itself, making the design and approval of studies particularly difficult (this also applies to pharmacological studies in pediatric ADHD) (40). However, in view of the significant health needs of children with ADHD that are not met, and the general importance of providing robust clinical evidence in pediatrics (41), it is urgently required to design studies that consider the complexities of research in such vulnerable study populations. In the following sections, we examine the ethical challenges of conducting research with tDCS in children with ADHD based on established frameworks of research ethics (16, 17) and ethical innovation [e.g., Ref. (18)], in order to develop recommendations on how to design studies that are ethical.

## Safety

The issue of safety in tDCS is often regarded to be marginal, since the applied electrical current is low when it comes to the potential of causing neuronal injury (21). Associated unintended effects of the procedure are mild headaches, itching, or weak burning on the spots where electrodes are placed (29). More severe harm might occur as a result of a failure of the equipment, although this is more likely to happen when used in nonclinical environments and especially from DIY systems outside of any medical oversight, which, unlike research-grade stimulation devices, do not provide built-in safety features (29).

The type and magnitude of reported adverse effects in the tDCS studies do not differ between children/adolescents and adults, and available evidence delivers no established risks specific to tDCS apart from those mentioned above. A recent German guideline includes tDCS for children and adolescents, thus mirroring recommendations for adult populations (42).

However, this assessment might be premature. Due to increased excitatory activity in young children, they could be more prone to seizures due to increased glutamate sensitivity, reduced glutamate clearance, and incomplete GABA-mediated inhibition in the developing brain (43), and this has to be monitored carefully.

Moreover, many studies in children and adolescents have used adult parameters without adjustment. Recently, it was shown that contrary to effects in adults (10), in a study with healthy children and adolescents, corticospinal excitability was found to increase, rather than decrease, following 1 mA cathodal tDCS. Various anatomic parameters change with age: thickness of the scalp and calvarium, scalp-to-brain distance, cerebrospinal fluid volume, and developmental changes in tissue architecture. Previous studies that performed current flow modeling in children suggested that a reduced tDCS current intensity is enough to produce the same peak brain electric fields in children compared with adults (44). Using realistic head modeling, Kessler and colleagues (45) demonstrated that lower applied current intensity (ca. 1 mA) may achieve brain current densities in young participants on average comparable to densities seen in adults to 2 mA current. Therefore, future studies have to consider that neuroanatomical differences of children’s brains might impact their stimulation protocols based on an adult population. Hence, the same tDCS parameters could have a larger impact in children and adolescents (46). This could lead to unexpected or even dangerous results if it misdirects plasticity or increases the likelihood of brain tissue lesions due to a thinner skull, resulting in higher peak current density in the child’s brain (47, 48).

Another concern of safety is the potential of tDCS to alter cognition in unintended ways. There is still limited evidence on this issue, but available findings suggest that stimulation of a certain part of the brain might improve some of the targeted brain activities, while impairing others at the same time (49). In ADHD, where the treatment is focused on, i.e., improving working memory, this could lead to a deterioration of functioning in other parts of the brain. The concern has thus been raised that stimulation aiming to enhance attention or working memory could have detrimental effects for cognition associated with creativity (50). Moreover, even within the construct of working memory, different parameters may be either improved or worsened by tDCS. For example, it has been demonstrated that some types of anodal tDCS can improve reaction time and performance variability but worsen error processing in adolescents with ADHD (24). Furthermore, it cannot be fully established whether changes induced by tDCS will be temporary or permanent. In the developing brain, there is a risk that changes might impact cognitive developmental trajectories in an undesirable way, particularly in view of the high plasticity of the developing brain. Some researchers have also raised concerns that the use of tDCS might be detrimental to a developing brain because it might alter cognitive functions, worsen the brain’s overall development or lead to interactions with pharmacological treatment (47).

## Benefits and Risks

Ethical acceptability of research studies in vulnerable groups rests on an assessment of the real benefits and risks. First of all, according to established ethical guidelines on biomedical research, the exposure to risk in research is ethically justifiable only if there is a social and scientific value of the research (17). This is the case in tDCS because there is a clear need that has not been met—current treatment alternatives for ADHD in children are not sufficient for treating all patients successfully. In addition, there might also be direct benefits to individual study participants who might find tDCS an effective treatment with fewer side effects compared to medication.

However, the assessment of potential benefits is complicated by the necessity to factor in the environmental, emotional, and other factors that might influence the tDCS data during a stimulation. Available research shows the significance of measuring the environmental and emotional factors—so-called state-dependent tDCS (51, 52); nevertheless, little is known about the extent to which those are considered during actual data collection. Another such factor is the brain’s state “wake vs. sleep,” since in children with ADHD, the application of tDCS during sleep had a beneficial impact on cognitive performance (27, 53). Documenting these factors systematically might require a more in-depth assessment prior to every stimulation session.

For any study on a vulnerable population to be ethically acceptable, usually, a positive ratio of benefits to risks is required, which is also difficult to assess in tDCS. To examine whether tDCS is an effective treatment, at least some study participants will have to forgo pharmacological treatment. In early stages of research, the assessment of an add-on effect (e.g., using designs such as “medication+tDCS vs. medication+sham”) could be sufficient. However, in the search for alternatives to standard treatments, research involving groups where the concomitant standard treatment is omitted is desirable.

ADHD medication might have side effects, but it has overall been proven to be effective and its mechanisms are well known. Thanks to long-term studies (54) and the knowledge of pharmacokinetics, it is much more feasible to anticipate possible adverse events. In contrast, the mechanisms of tDCS are less well explored, contributing to the list of “unknowns” in tDCS. Lack of evidence regarding the long-term effects of stimulation also complicates assessing the risk–benefit ratio.

Finally, the question of risk assessment should include both temporary and potentially permanent (unwanted) changes in cognition. For instance, if changes occurred only for a limited time, this might be ethically acceptable; however, any risk of permanent changes would affect the ethical acceptability of the risk–benefit ratio significantly.

Overall, in addition to considering standard inclusion/exclusion criteria, the “unknowns” in tDCS in pediatric ADHD mandate the individual risk assessment of every participant at the time of inclusion, for example, regarding the potential withdrawal of medication. It is also important to minimize and monitor any findings regarding unwanted changes in cognition closely, and to conduct longer follow-up. Finally, it must be clearly communicated, especially to study participants and their parents, that some risks have not yet been fully mapped/understood.

## Information and Consent

A long-standing principle of research ethics requires that research is only performed if informed consent has been given. In case of pediatric ADHD, research needs to be conducted with a study group that is vulnerable in two respects.

First, research occurs in minors, a group that by definition lacks both full functional and full legal capacity to provide informed consent. For this reason alone, children as research participants are regarded as a group that warrant special protection (55).

Second, subjects in pediatric ADHD research are vulnerable in that they have a neuropsychiatric disorder, which might contribute to a lower or fluctuating capacity to understand and consent to research, thus impacting on their autonomy and decision-making. Furthermore, the high comorbidity of ADHD and learning difficulties (56) might impact patients’ comprehension, ability to read a written material, and to understand the assent to treatment. This is compounded by the fact that the onset of ADHD is usually at a young age, making it particularly difficult to gain an adequate understanding of the research and providing adequate consent.

In populations that cannot (yet) consent themselves, surrogate consent by legal guardians—in the case of children, usually parents—must be issued. In this process of decision-making, depending on a child’s age and maturity, their perspective should also be considered (16, 17). Usually, there is a requirement of seeking the child’s assent (a form of approval below the standard of consent). Regulation varies across countries in establishing the age thresholds for assent [for the EU overviews, see Ref. (57)]. As an alternative to age thresholds, more personalized approaches to improving children’s understanding and gaining their approval for study participation have been proposed (58).

In tDCS research in children with ADHD, the provision of information to study participants and their parents is further complicated by the limited knowledge of potential risks of this technique. In addition to the aforementioned “unknowns” related to the overall mechanism and effect of stimulation, long-term risks, and the lack of translational studies from adults to children, there is also a need for clear dosing guidelines (59). All this results in several difficulties when designing tDCS studies for children with ADHD, including how to determine which doses are safe, how to develop a framework for establishing informed consent in children and their carers, and how to implement an efficient system for monitoring and reporting adverse effects during and following the brain stimulation in minors (see also the discussion above under Safety).

Communicating these complex considerations appropriately to parents and children is a major challenge for translational research on tDCS in pediatric ADHD. Guidance on how to enable an informed decision through clear and transparent information and communication that is also attuned to the particular needs of the population, should be developed.

## Labeling Problems

TDCS is usually described as noninvasive. This relates to the fact that the technique is applied through the skin, without any surgical procedures. Furthermore, being a relatively safe procedure with minimal side effects in adults contributes to this characteristic [see Regulation (EU) 2017/745]. However, it has been argued that using the term “noninvasive” with regard to tDCS might be misleading (60). It should therefore be explained why applying a direct current across the skull with effects on brain function can be considered noninvasive in case of tDCS. Otherwise, there is the danger that participants and surrogate decision makers may underestimate or overestimate the effects of the stimulation. On the other hand, it is important that tDCS is distinguished from procedures that require, e.g., surgery, such as DBS, or general anesthesia, such as electroconvulsive therapy (ECT) (especially in view of the stigma of ECT still present, due to its history) (61). While both of these also involve electrical stimulation, they have significantly higher risks than tDCS.

One way around the challenge of labelling would be to avoid using the word “noninvasive” in study information materials altogether, as it might lead to confusion, which eventually might undermine research practices in this area. Alternatively, by presenting tDCS and its characteristics in comparison with other procedures, participants and carers could decide for themselves whether the degree of invasiveness of tDCS is acceptable to them.

## Nonmedical, do-it-Yourself Transcranial Direct Current Stimulation and Home Devices

The use of ADHD medications for nonmedical purposes has been discussed in the literature, especially with regard to so-called “cosmetic psychopharmacology” (62–64). This involves individuals without a diagnosis of ADHD using ADHD drugs for “enhancing” purposes, such as better concentration, focus, or alertness at work or in school (62, 63). There is some anecdotal evidence (e.g., YouTube videos and online communities) that people use brain stimulation—often through DIY tDCS kits—for similar purposes (33). This issue thus requires attention.

The ethical implications of applying tDCS for enhancement purposes in minors have not yet been sufficiently explored [but see Ref. (36)]. With other interventions, there is consensus that in children, the use of neuroenhancement should be strongly discouraged due to the fact that children’s’ brains are still developing and any treatment that is not medically necessary could pose risks of harm (65). And indeed, home tDCS use might result in a different current flow in pediatric brains (45, 66). This, in turn, leads to an important challenge when researching tDCS in children with ADHD, particularly when there is a goal to develop home application kits. Home devices might enable more successful therapeutic application and make the treatment available to a wider group of patients. Safety issues of home use of tDCS have been discussed in the literature (21, 29), however, there is a general scarcity of research on home-based tDCS (67). Additionally, the potential for misuse, e.g., for enhancement purposes, and whether researchers are obliged to curb this, should be further addressed.

## Conflicts of Interest and Commercialization

Finally, as with any novel technology, there is commercial interest in developing devices and applications of tDCS for pediatric populations. This is not unethical as such, but it does come with potential conflicts of interest. As with all research, the latter must be avoided, and researchers should be transparent regarding the support of, e.g., manufacturers in developing stimulation applications, particularly for home settings. The low cost and relatively easy application of tDCS and the current lack of regulation might create a possibility for industry to market devices directly to consumers and healthcare professionals who lack any in-depth knowledge of tDCS.

## Discussion and Recommendations

The analysis of ethical issues above highlights the need for a more rigorous ethical framework for pediatric tDCS studies. So far, this has not developed beyond calls for researchers to be aware of their ethical responsibility (29), or to exert extreme caution (59). Here, based on established principles of research ethics as well as the ethical analysis above, we attempt to provide clinicians and researchers with some initial and practical recommendations on how to conduct ethical research with pediatric tDCS. These include recommendations on transparency, informed consent and assent, risk assessment, monitoring and reporting, public communication, and enhancement/home use. Our focus is on research with children with ADHD, as there are both an ethical imperative to address needs in this population that have not been met and a tension of “great needs matching great uncertainty” (68). The framework developed below, however, could be applied to a broader range of studies on tDCS and neuropediatric disorders (see **Table 1**). Obviously, all research on tDCS in pediatric ADHD must correspond with established principles of research ethics. Thus, in our recommendations, we do not repeat every requirement for research to be ethical [but see Refs. (16, 17)]. Instead, we focus on those issues that are particular to tDCS, or that warrant special attention from researchers working with this technology in children. We also do not claim that the guidelines derived from the framework are complete. We offer these recommendations to all colleagues involved with tDCS for neuropsychiatric disorders in children, to the professional associations involved in the field, and to the wider professional community as a starting point for discussing and developing more comprehensive guidelines for future practice. We hope that they can serve as a first step to ensure research in this promising area is ethical, and the translation into the clinic successful.

**Table 1:** Ethical framework and recommendations for ethical research on transcranial direct current stimulation (tDCS) in pediatric populations with attention deficit hyperactivity disorder (ADHD)*.

Transparency	- Parents and their children with ADHD are especially vulnerable groups. The provision of information should be transparent in order to allow decision-making based on comprehensive information. This is especially important in view of several “unknowns” in tDCS. It should also strike the balance between raising unwarranted hopes on the one hand and scaring children and their parents away from participating in research on the other. - Potential conflicts of interest should be avoided, and any involvement of commercial actors should be described transparently. - Researchers should be encouraged to publish and disseminate any negative results, failed experiments, issues in optimization, etc., to avoid redundant research. - Researchers should provide anonymized raw data, and assessment and access to the standardized set of context data to identify conditions of tDCS to colleagues in the field.
Adjusted informed consent and assent	- Apart from standard information about the potential (low) risks of the tDCS application, honest information about the current state of research should be added to information materials. - Discussion of the unknowns and uncertainties of tDCS in children, particularly with a view to risks, should take place prior to application. - Referring to tDCS as “noninvasive” should either be avoided entirely in information materials, or clear information on why this method is perceived as noninvasive should be provided. - Information about the potential conflicts of interest should also be listed at this stage. - In view of the current state of uncertainty, any information materials should be regularly assessed and updated, in view of emerging information and evidence. - Information materials should be tailored to the population and also use nonstandard formats such as videos, etc. - A clear statement that participants (children) might not benefit from experimental use of tDCS should be included in information material.
Risk-assessment, monitoring and reporting	- Detailed and specific monitoring and reporting plans as well as risk minimization plans should be part of every study with tDCS in children with ADHD, particularly where withdrawal of medication is necessary in some study groups. - Individual risk assessment should be performed for each patient considered for inclusion; overall fit with inclusion/exclusion criteria developed for study groups is not sufficient. - Changes in cognition should be monitored especially closely, e.g., with a view to changes in creativity; this might require developing novel assessment tools. - Detailed information about comorbidities and ADHD should be collected and reflected on how it might impact the treatment with tDCS in children. - Interaction with medication (for ADHD and other conditions) should be particularly closely followed and reported. - Studies should, whenever possible, investigate how risks and effects differ in different cohorts: ages, stages of ADHD, etc. - Monitoring of environmental and state-dependent effects and potential influences should be performed (e.g., emotional events that occupy the participant’s mind and might influence the data).
Responsible public communication	- Hype should be avoided when presenting studies and results; instead, honesty and transparency should be emphasized. - Researchers should work closely with manufacturers on how the information about the devices is presented in an appropriate manner. - Researchers should cooperate with regulatory agencies on developing guidance how to prevent misuse of tDCS - Researchers should address issues of enhancement and nonclinical use in children and adolescents openly and educate the public about risks thereof.
Enhancement and home use	- Researchers should educate themselves about available home applications and nonmedical, enhancement use of tDCS. - Researchers should discuss nonmedical use with families openly and in a nonjudgmental manner. They should provide information about the risks unmonitored, nonclinical use of tDCS can have on children and adults. This might require developing specific information materials. - In case there is a possibility of tDCS to be used as enhancement—e.g., in home devices and by other family members, researchers should articulate the potential risks and signal that they are available to discuss the ethical issues related to cognitive enhancement throughout the course of a study, and beyond.

tDCS, transcranial direct current stimulation; ADHD, attention deficit hyperactivity disorder.

*In addition to standard requirements of research ethics.

## Author Contributions

AS: substantial contributions to the conception or design of the work, drafting the work or revising it critically for important intellectual content. AP-K: substantial contributions to the content development. VM: substantial contributions to the content development. KK: substantial contributions to the content development. RN: substantial contributions to the content development. CF: substantial contributions to the content development, revising it critically for important intellectual content. MS: substantial contributions to the content development, revising it critically for important intellectual content. AB: substantial contributions to the conception or design of the work, revising it critically for important intellectual content.

## Funding

This project has received funding from the European Union’s Horizon 2020 research and innovation program under grant agreement no. 731827. This text reflects only the authors’ views and the Commission is not liable for any use that may be made of the information contained therein.

## Conflict of Interest Statement

RN works as a full-time employee for Neuroelectrics.

The remaining authors declare that the research was conducted in the absence of any commercial or financial relationships that could be construed as a potential conflict of interest.
